# The Effect of Different Types of Walking on Dual-Task Performance and Task Prioritization among Community-Dwelling Older Adults

**DOI:** 10.1155/2014/259547

**Published:** 2014-11-23

**Authors:** Maayan Agmon, Einat Kodesh, Rachel Kizony

**Affiliations:** ^1^Department of Nursing, Faculty of Social Welfare & Health Sciences, University of Haifa, Mount Carmel, 31905 Haifa, Israel; ^2^Department of Physical Therapy, Faculty of Social Welfare & Health Sciences, University of Haifa, Mount Carmel, 31905 Haifa, Israel; ^3^Department of Occupational Therapy, Faculty of Social Welfare & Health Sciences, University of Haifa, Mount Carmel, 31905 Haifa, Israel; ^4^Department of Occupational Therapy, Sheba Medical Center, 5262000 Tel Hashomer, Israel

## Abstract

*Background*. The ability to safely conduct different types of walking concurrently with a cognitive task (i.e., dual task) is crucial for daily life. The contribution of different walking types to dual-task performance has not yet been determined, nor is there agreement on the strategies that older adults use to divide their attention between two tasks (task prioritization). *Objectives*. To compare the effect of walking in three different directions (forward, backward, and sideways) on dual-task performance and to explore the strategies of older adults to allocate their attention in response to different motor task demands. *Design*. A cross-sectional study. *Subjects*. Thirty-two (22 female) community-dwelling older adults (aged 72.7±5.7 years). *Methods*. Subjects randomly conducted single and dual task: walking to three directions separately, cognitive tasks separately, and combination of the two. *Results*. Walking forward was the least demanding task, during single (FW < BW, SW) (*P* < .001) and dual tasks (FW < BW < SW) (*P* < .001). The calculation of DTC revealed the same pattern (*P* < .001). DTC of the cognitive tasks was not significantly different among the three walking types. *Conclusions*. The decline mainly in the motor performance during dual task indicates that participants prioritized the cognitive task. These findings challenge the “posture first” paradigm for task prioritization.

## 1. Introduction 

The ability to safely perform different types of walking such as forward (FW), sideways (SW), and backward (BW) is crucial for maintaining daily functioning. When walking, people often engage in a second task (dual tasking: DT) such as talking on the phone or simultaneously talking when walking sideways between two rows of seats at a theater. To perform DT efficiently, attention resources must be divided appropriately between the cognitive and the postural (e.g., walking) tasks [1]. A variety of factors have been found to exert a major effect on an individual's ability to divide attention between two tasks. Among them are intrinsic factors such as age, executive function, and physical status and external factors such as the tasks' difficulty [2, 3]. The ability to perform DT deteriorates with aging [4], which results in higher rates and risk of falls [5] and functional decline [4, 6].

Several studies have focused on the effect of the cognitive task component on DT [7]; however, the effect of different motor tasks on DT is not well established [8]. Recently Simoni and colleagues [8] compared the effect of two different motor tasks (treadmill versus over-ground walking) on cognitive performance in older adults and found that treadmill walking did not affect the latter, while with over-ground walking both tasks were affected. Their study provided new insights into the relative contribution of the motor task to DT performance. However, it did not present dual-task cost (DTC), which is the change in DT compared with single task (ST) performance of the same task [9], for any of the tasks. Consequently, the study did not contribute to our understanding of task prioritization. In addition, studies comparing FW, BW, and SW [10–12] are limited more so with an additional cognitive load (i.e., DT).

A major intrinsic factor that influences the ability to perform DT safely in daily life is task prioritization, namely, how a person allocates attention to the two tasks [13]. The paradigm of task prioritization strategies has been challenged over the years. Traditionally, the “posture first” strategy, holding that young and older adults alike prioritize the postural over the cognitive task as part of survival and safe adaptation, was the leading model [14]. However, Yogev-Seligmann and colleagues [15] recently expanded this model by adding the “cognitive first” strategy as an equal substitute for the “posture first” strategy. This model postulates the interplay between postural reserve and hazard estimation as significant intrinsic factors contributing to the selection of the task prioritization strategy. In keeping with this model, Liston et al. (2014) [1] demonstrated that older adults did not prioritize postural tasks while multitasking, in contrast to younger adults who did adhere to the “posture first” paradigm. However, whether older adults can flexibly adjust the prioritization strategy in response to various motor demands is not yet clear. To evaluate task prioritization, DTC [9] has to be calculated for the cognitive and the motor task separately. DTC is calculated by this formula: ST performance minus DT performance divided by ST performance, multiplied by 100 to be expressed as a percentage:

(1) $$\begin{array}{c} \left(\frac{\left(\text{ST}-\text{DT}\right)}{\left(\text{ST}\right)}\right)*100 \end{array}$$

(see [16]).

Currently, there is a need to further elucidate the role of the motor task in DT performance and to analyze task prioritization strategies corresponding to different motor tasks in older adults. The objectives of the current study were to (1) compare the effect of different motor tasks (i.e. FW, SW, and BW) on DT performance and (2) to compare attention allocation strategies during different motor conditions of walking types (FW, SW, and BW).

## 2. Methods

### 2.1. Participants

Thirty-two community-dwelling older adults were recruited by the snowball sampling method. Inclusion criteria were (a) aged 65 years and older, (b) able to walk independently, (c) can speak, understand, and read Hebrew or English, and (d) achieve a Montreal Cognitive Assessment (MoCA) [17] score ≥ 21. Exclusion criteria were (a) any active and untreated symptomatic illness that might limit ability to complete the tests, (b) neurologic or musculoskeletal diagnosis, (c) severe orthopedic restrictions that might affect walking, and (d) significant hearing or vision loss. The study was approved by the Institutional Review Board of the University of Haifa and written informed consent was obtained from all participants.

### 2.2. Procedure

This study used a cross-sectional design. All participants performed FW, BW, and SW on a flat surface marked at the beginning and at the end for one minute without a cognitive task (ST) and with a cognitive task (repeatedly subtract 3 from a random number between 100 and 250) as a DT. Participants were also asked to perform the same cognitive subtraction task while seated. The order of single and dual tasks was random. Participants were instructed to conduct both tasks to the best of their ability; no instructions were given regarding task prioritization. Motor task performance was measured as the distance walked in one minute in each walking condition; cognitive task performance was measured as the number of correct responses in one minute in each condition.

#### 2.2.1. Measurements of Cognitive and Dynamic Postural Control Abilities

MoCA is a screening test developed to detect mild cognitive impairments. The test screens cognitive abilities in 7 domains (e.g., executive functions and memory) and scores range between 0 and 30. The test was found reliable and sensitive to detect mild cognitive impairment [17]. A threshold of 24 has 96% negative predictive value and 47% positive predictive value in the detection of cognitive impairment [18].

Balance Evaluation Systems Test (Mini-BESTest) [19] is a 14-item performance-based measure of balance disorders, shown to be reliable and valid for chronic stroke. The tasks test different balance subsystems, including responses to external perturbations, anticipatory postural adjustments, gait stability, and sensory orientation. Scores range from 0 to 28. Test retest reliability for the Mini-BESTest total scores was 0.96, interrater reliability was 0.98 [20].

Timed-Up-and-Go (TUG) [21] measures the time taken by a participant to rise from a standard chair, walk 3 meters, turn, walk back, and sit down on the chair.

### 2.3. Statistical Analysis

Data was analyzed using SPSS 18.0 for Windows. The Shapiro-Wilk test results showed that three variables (distance walked during SW and BW as single tasks and during BW as a dual task) were not normally distributed. Therefore, for comparison of single and dual motor tasks, Wilcoxon signed ranks tests were used. For FW the distribution was normal, so a paired *t*-test was used to compare ST and DT. Comparisons within ST and DT motor task, for the three walking types, were analyzed using the nonparametric Friedman test, followed by ad hoc analysis using Wilcoxon signed ranks tests between each pair of walking types. DTCs were distributed normally; thus, comparison of performance of the cognitive tasks as well as motor and cognitive DTC in the three walking types was analyzed using ANOVA repeated measures, followed by contrast analysis when the overall model was significant. Effect sizes (ES) for all comparisons between the three walking directions under ST and DT were calculated for nonparametric tests based on Field, 2013 [22].

## 3. Results

### 3.1. Participants

Twenty-two females and ten males aged 72.7 ± 5.7 years participated. Demographic characteristics and results of balance and cognitive measurements are presented in Table 1. Overall, the sample was comprised of high-functioning older adults (both in ADL and IADL) with normal gait speed (mean TUG 7.6 seconds) and normal balance. The sample is highly educated with normal cognitive function according to MoCA scores [18]. Three participants reported falls during the past year.

#### 3.1.1. Differences in Performance between Single and Dual Task Conditions in Each of the Three Walking Types

Significant differences were found between distances walked during single and dual tasks in all walking types: BW, *z* = −4.56, *P* = .0001; SW, *z* = −4.94, *P* = .0001; FW, *t*_(31)_ = 7.04, *P* = .0001 (see Table 2). In addition significant differences were found between performances of the cognitive task during single and dual tasks in all walking types: BW, *t*_(31)_ = 4.46, *P* = .0001; SW, *t*_(31)_ = 4.91, *P* = .0001; FW, *t*_(31)_ = 4.79, *P* = .0001.

#### 3.1.2. Differences in Performance of Single and Dual Tasks between the Three Walking Types

Distance walked during FW, in both single and dual task conditions, was significantly longer than distance walked during SW (ES = 0.85; ES = 0.87, resp.) and BW (ES = 0.87; ES = 0.87, resp.). Distance walked during BW was significantly longer than during SW in dual task condition only (ES = 0.35). Effect size of the difference between SW and BW under single task was 0.06.

No significant differences were found in the performance of the cognitive task during the three walking types.

#### 3.1.3. Differences in DTC between the Three Walking Types

Significant differences were found between the DTC of the distance walked in the three walking tasks with subtraction (*P* = .001). The DTC during FW was significantly lower than the DTC during SW and BW. The DTC during BW was significantly lower than the DTC during SW. No significant differences were found between the DTCs of the cognitive task in the three walking types.

## 4. Discussion

This study investigated the effect of motor task (i.e. three walking types) on DT performance in older adults and explored whether task prioritization was consistent or varied in response to the difficulty of the motor task. As expected, the main findings indicate that FW was the least demanding task, as reflected in the distance walked during single and dual tasks, as well as in the DTC of the motor task. SW was more demanding than BW but this was only evident in the distance walked during the DT and in the DTC of the motor task. However, cognitive performance and DTC of the cognitive tasks did not differ significantly in the three walking types, indicating that regardless of the motor challenges, participants prioritized the cognitive task.

Several potential explanations have been suggested as underlying mechanisms for the differences between FW and BW [10–12], however reports about SW are very limited. BW requires higher energy consumption than FW [23], which is generally characterized by a decreased walking speed due to limited ability to see the direction of the progress [11]. It also requires a different kinematic pattern and allows a smaller range of motion in the hip, resulting in shorter stride length [11]. The main reasons for the significantly shorter distance walked in SW than in FW may stem from similar mechanisms. In FW energy consumption was found lower than in SW [24]. Furthermore, in SW participants in the current study walked using a planar gait which involves a phase of straight standing. This type of SW requires mainly mediolateral postural control, which is considered a highly challenging task for older adults [25]. In contrast, FW and BW require mainly anterior-posterior postural control [11], which is deemed less demanding. The distance walked in BW was significantly longer than in SW, but this was only apparent when cognitive load was added (i.e., DT). The combination of increased cognitive load and the mediolateral postural control demanded in SW seemingly resulted in higher DTC than in BW. This finding is in line with previous studies demonstrating that DT interference is higher when task combination is more challenging [7]. An alternative explanation might be that to ensure the participants' safety, one of the researchers walked behind the subjects and cued them before turns during BW. This kind of supervision may not accurately represent BW in real-world situations and may confound the differences between SW and BW. Nevertheless, the results regarding the differences between SW and BW should be interpreted with caution due to the small sample size, which may account for the lack of significance between the two walks under single task condition.

The results of the current research support findings of previous studies showing a decline in performance of both motor and cognitive tasks under DT conditions while walking over-ground [8]. The current study further elaborated this finding by showing, for the first time, the effect of three types of over-ground walking on DT performance. Moreover, the analysis of differences in DTC of both motor and cognitive tasks across the three walking types sheds light on the strategies used by the participants to cope with the increased motor and cognitive demands. Our findings show that DTC of the motor task increased when the motor aspect was more challenging (i.e., FW < BW < SW); however this was not accompanied by an increase in the cognitive DTC. These findings contradict the “posture first theory” [21]. Yogev-Seligmann et al. [15] suggested that personal characteristics such as risk judgment and postural stability might influence the prioritization strategy that the individual would unconsciously use. In the current study all participants were active community-dwelling older adults with normal postural control and hence could prioritize the cognitive task while walking safely, without regard to the difficulty of the walking condition. Future studies should address which task conditions as well as personal characteristics influence the strategy that the individual would execute.

## 5. Conclusion

Our findings could contribute to a better understanding of motor control and the DT paradigm as well as further illuminate prioritization patterns among older adults. Like every study, this study has some limitations, such as (1) small, predominantly female sample that was recruited by the snowball method and (2) limited generalizability to individuals who are not relatively healthy and highly functioning. Future studies should further explore the way personality interacts with task characteristics to determine which strategy will be executed. In addition, a longitudinal study, including the calculation of DTC of both tasks, should be carried out to determine whether prioritization patterns have a predictive value for future falls. Moreover, analysis of spatial and temporal gait parameters and gait variability may better reflect the difference between walking directions. In addition, the question whether dual-task training in one direction transfers to other direction should be explored. Finally, stronger sampling methodology and more heterogeneous sample that includes older adults with cognitive and physical impairments may contribute to the generalizability of the findings.

The current study also may have several clinical implications that could inform protocols for DT training aiming at a fall-prevention program. These include the need for specific instructions during training in order to focus attention on one task or the other and to measure performance of both tasks during dual-task training in order to prevent prioritization of one task at the expense of the other. In addition, the graded protocol should start from forward walking followed by backward and sideways walking.

## Figures and Tables

**Table 1:** Sample characteristics (*N* = 32).

Measure	Mean ± SD
Age (years)	72.7 ± 5.72
Education (years)	14.5 ± 2.70
Montreal Cognitive Assessment (MoCA) (0–30)	25.4 ± 2.37
Balance Evaluation Systems Test (Mini-BEST) (0–30)	25.3 ± 2.39
Timed-Up-and-Go (TUG) (seconds)	7.6 ± 1.78

**Table 2:** Comparisons between single and dual tasks and dual-task costs across walking types.

Task	Forward	Backward	Sideways		
ST Vs DT motor^a^	Median (interquartile range)			*χ* ^2^_(df=2)_	Wilcoxon signed ranks test

Single task (distance; meter)	71.50 (63.00–82.63)	43.00 (35.63–50.50)	37.75 (31.25–54.50)	41.44^**^	*F* > *B*, *S* ^**^
Dual task (distance; meter)	55.50 (46.63–65.88)	27.00 (18.75–39.63)	26.00 (19.00–30.75)	50.45^**^	*F* > *B* > *S* ^*^

ST Vs DT cognitive^a^	Mean ± SD			*F*_2,30_	Contrasts

Single task (# of correct responses)	31.75 ± 10.67	31.75 ± 10.67	31.75 ± 10.67	NA	NA
Dual task cog. (# of correct responses)	24.81 ± 8.43	27.38 ± 10.70	25.22 ± 9.18	3.23	NA

DTC of motor and cognitive tasks	Mean ± SD				

DTC distance, mean ± SD	21.44% ± 16.55	29.86% ± 21.50	37.52% ± 18.32	8.69^**^	*F* < *B* < *S* ^*^
DTC cog., mean ± SD	15.91 ± 22.43	11.07 ± 15.60	15.87 ± 19.52	1.11	NA

^a^Significant differences were found between ST and DT for motor and cognitive tasks in all three walking types; *P* = .0001.

^*^ *P* < .05, ^**^ *P* < .001.

## Abbreviations

DT: Dual task
ST: Single task
FW: Forward walk
BW: Backward walk
SW: Sideways walk
DTC: Dual-task cost.
